# Nitrogen-Doped Carbon Dots for “green” Quantum Dot Solar Cells

**DOI:** 10.1186/s11671-016-1231-1

**Published:** 2016-01-19

**Authors:** Hao Wang, Pengfei Sun, Shan Cong, Jiang Wu, Lijun Gao, Yun Wang, Xiao Dai, Qinghua Yi, Guifu Zou

**Affiliations:** College of Physics, Optoelectronics and Energy & Collaborative Innovation Center of Suzhou Nano Science and Technology, Soochow University, Suzhou, 215006 People’s Republic of China; Department of Electronic and Electrical Engineering, University College London, Torrington Place, London, UK

**Keywords:** Carbon dots, Nitrogen-doped, Quantum dot solar cells

## Abstract

Considering the environment protection, “green” materials are increasingly explored for photovoltaics. Here, we developed a kind of quantum dots solar cell based on nitrogen-doped carbon dots. The nitrogen-doped carbon dots were prepared by direct pyrolysis of citric acid and ammonia. The nitrogen-doped carbon dots’ excitonic absorption depends on the N-doping content in the carbon dots. The N-doping can be readily modified by the mass ratio of reactants. The constructed “green” nitrogen-doped carbon dots solar cell achieves the best power conversion efficiency of 0.79 % under AM 1.5 G one full sun illumination, which is the highest efficiency for carbon dot-based solar cells.

**Electronic supplementary material:**

The online version of this article (doi:10.1186/s11671-016-1231-1) contains supplementary material, which is available to authorized users.

## Background

Among the third-generation photovoltaics, quantum dot solar cells (QDSCs) are emerging as a promising candidate due to the unique and versatile characteristics of quantum dots (QDs) including tunable band gap and high absorption coefficient [1–4]. Typically, low-band gap metal chalcogenide (CdS, CdSe, CdTe, PbS, PbSe, CuInS_2_, etc.) QDs are widely used as sensitizers in QDSCs [5–10]. The QDSCs have been well developed, but they contain highly toxic metals (including Cd, Pb, and In). Hence, the environmentally friendly alternatives (“green” materials) are extremely needed and welcomed to the fabrication of solar cells.

Due to the unique optical and electronic properties, water solubility, excellent biocompatibility, low toxicity, and robust chemical inertness [11–15], carbon dots (CDs) as an emerging carbon-based nanomaterial have been widely researched and applied in many fields [16–22]. One of the CDs applications is tentatively used as sensitizers for QDSCs [23–26]. For instance, Mirtchev et al. [24] have prepared water soluble CDs as sensitizers for QDSCs achieving power conversion efficiency (PCE) of 0.13 %. Zhang et al. [25] have also fabricated 0.13 % PCE of QDSCs based on nitrogen-doped CDs (NCDs). Up to now, the low short-circuit current density (*J*_sc_) might be the main factor limiting the efficiency of the cells in comparison with the open-circuit voltage (*V*_oc_) and fill factor (FF) of metal chalcogenide-based QDSCs. Narrow light-absorption and diverse trap states result in the extremely low *J*_sc_ of CD-based QDSCs. Meanwhile, CDs’ light-absorption is centered at ultra-violet region and very weak at visible region. Because the existence of trap state defects with different energy levels has been demonstrated by their excitation-dependent fluorescence [27], partially photoinduced carriers will be recombined by CDs rather than being injected into the transporting media. Accordingly, the PCE of QDSCs is very low. Much effort still needs to improve the practicable sensitizers for QDSCs.

In this study, we have developed NCDs to build up the “green” QDSCs with enhanced efficiency. The NCDs were prepared by direct pyrolysis of citric acid and ammonia. Experimental results show that the NCDs’ excitonic absorption depends on the N-doping content modified by the mass ratio of reactants. The NCDs with optimal excitonic absorption were used as sensitizers in the QDSCs. The constructed NCD solar cell achieves the best PCE of 0.79 % under AM 1.5 G one full sun illumination. It is noting that the obtained efficiency is the highest one in the reported solar cell-based CDs.

## Methods

### Synthesis

**Scheme 1:**
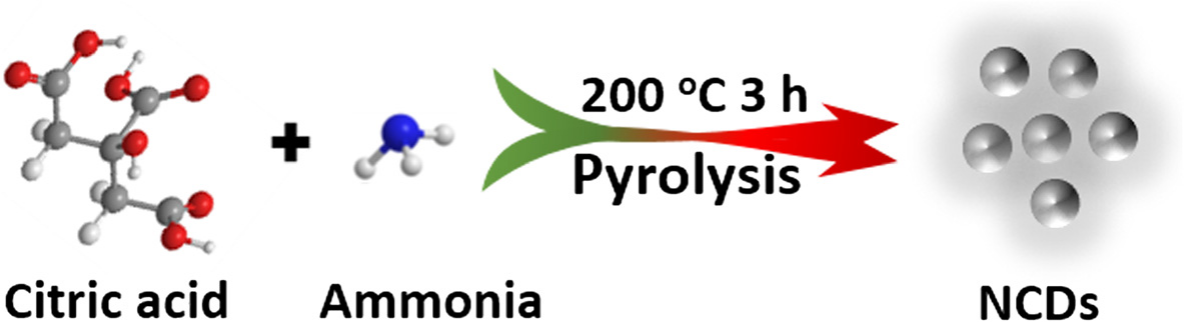
Preparation of NCDs via direct pyrolysis of citric acid and ammonia

The NCDs were prepared by a direct pyrolysis method. As shown in Scheme 1, the NCDs are prepared by direct pyrolysis of citric acid (CA, Alfa Aesar) and ammonia (Sinopharm Chemical Reagent Co.) mixture similar to the previous report [28]. Typically, CA (4 g) and certain amount of ammonia are dissolved in distilled water (10 mL) with stirring for 30 min. Then, the prepared solution is transferred into a porcelain boat and then heated in air at 200 °C for 3 h with a heating rate of 10 °C/min. The obtained carbogenic product is mixed with acetone (40 mL, Sinopharm Chemical Reagent Co.) by ultrasonic. The supernatants containing NCDs are collected after centrifugation at 10,000 rpm for 15 min. Then, the acetone is removed by rotary evaporation, and solid-state NCDs are collected for further characterization and usage.

### Fabrication of Solar Cell

The NCD-sensitized TiO_2_ photoanodes are fabricated according to the previous report [29]. The cleaned fluorine-doped tin oxide (FTO) glasses are immersed in 40 mM TiCl_4_ aqueous solution at 70 °C for 30 min and washed with water and ethanol. A 20-nm-sized TiO_2_ paste is deposited on the FTO glass plate by doctor blade printing technique and then dried at 125 °C for 5 min. The scattering layer of 200-nm-sized TiO_2_ paste is coated on the top of the first TiO_2_ layer, followed by sintering in air at 500 °C for 30 min. After cooling to 95 °C, the TiO_2_ film is immersed in the NCQD acetone solution at room temperature for 2 h. The NCD-sensitized TiO_2_ is washed with anhydrous ethanol and dried with nitrogen stream. The solar cells are fabricated by sandwiching gel electrolytes between a NCD-sensitized TiO_2_ electrode and a Pt counter electrode, which are separated by a 25-mm-thick hot-melt ring (Surlyn, Dupont) and sealed by heating. The electrolyte injection hole on the thermally platonized FTO counter electrode is finally sealed with Surlyn sheet and a thin glass by heating.

### Characterization and Measurement

The morphology of the materials is investigated by transmission electron microscopy (TEM, FEI Tecnai G-20) and atomic force microscopy (AFM, Asylum Research MFP-3D-BIO). Fourier transform infrared (FT-IR) spectra are obtained on a Bruker VERTEX70 FT-IR spectrometer ranged from 4000 to 400 cm^−1^. X-ray photoelectron spectra (XPS) are acquired with a Japan Kratos Axis Ultra HAS spectrometer using a monochromatic Al Kα source. The crystallinity of NCDs were characterized by Raman spectroscopy (HORIBA Jobin Yvon HR800) using 514-nm laser as the excitation source. The UV–visible absorption spectrum and diffuse-reflectance spectra are recorded on a UV2501PC (Shimadzu). The photoluminescence (PL) spectra are recorded on a Fluoromax-4 spectrofluorometer (HORIBA JobinYvon Inc.) equipped with a 150 W of xenon lamp as the excitation source. Photocurrent–voltage measurements of solar cells (Keithley2440 sourcemeter) are obtained by using a solar simulator (Newport) with an AM 1.5 G filter under an irradiation intensity of 100 mWcm^−2^ (550-W Xe source, Abet). The light intensity is calibrated using a standard silicon photovoltaic solar cell. The active cell area is 0.25 cm^2^. All of the measurements above are performed at room temperature.

## Results and Discussion

**Fig. 1:**
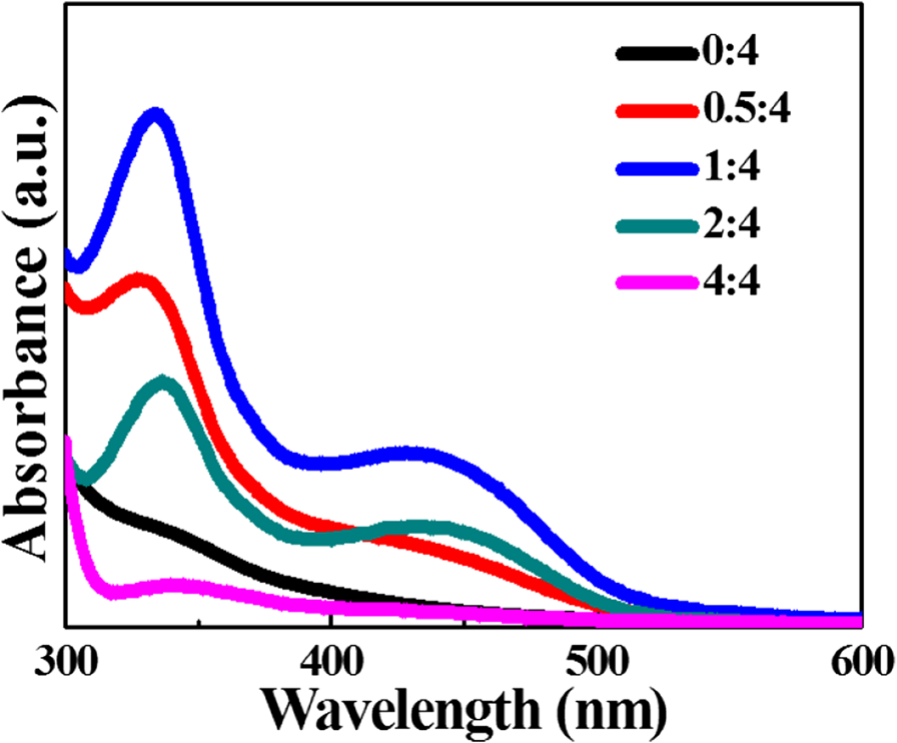
UV–vis spectra of NCDs from 4 g CA and different amounts (0, 0.5, 1, 2, and 4 g) of ammonia

The UV–vis spectra of NCDs from different reactant mass ratios show the NCDs’ excitonic absorption strongly depends on their N-doping content (Fig. 1). Our results demonstrate that the N-doping content is easily modified by the mass ratio of reactants. When the mass ratio of the reactants ammonia and CA is around 1: 4, the NCDs possess the optimal visible absorption. The excessive ammonia might happen strong chemical reaction with overheated NCDs resulting in excessive amount of water loss and the destruction of NCDs surface. To explore the effect of the mass ratio on the N-doping level of the NCDs, the element constitutes of the NCDs have been analyzed in Table 1. The N-doping content increases with the increasing mass ratio of ammonia and CA. Nevertheless, it changes slight when the mass ratio further increases over 4:1.

**Table 1:** Relative elemental analysis of the CDs with different N-doping levels by XPS analysis

Elemental/atomic (%)	C	O	N
N-free	78.4	21.6	0
0:4	74.8	22.6	2.6
1:4	67.5	22.5	10.0
2:4	69.7	21.1	9.7
4:4	71.5	19.2	9.3

**Fig. 2:**
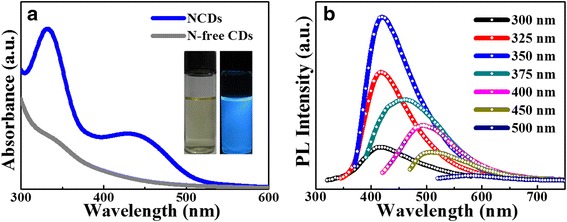
**a** UV–vis spectra of NCDs and N-free CDs. *Insets* are the photographs of NCDs aqueous solution under day light (*left*) and UV lamp irradiation (*right*). **b** PL spectrum of NCDs with excitation of different wavelengths recorded from 300 to 500 nm

The optical properties of optimal NCDs were further studied in Fig. 2. Compared with N-free CDs, the optimal NCDs exhibit not only much higher absorption peak around 335 nm but also more broad absorption band extending to 550 nm (Fig. 2a). In general, the absorption peak around 335 nm corresponds to n–π* transition of C=O bonds while the visible absorption band is attributed to the amino groups on the surface of NCDs [30]. The expanding absorption to 550 nm indirectly explains nitrogen-doping CDs. In addition, NCDs also exhibit excellent solubility with bright blue emission under UV lamp (365 nm) irradiation (inset of Fig. 2a). With the excitation wavelength increasing from 300 to 500 nm in Fig. 2b, the emission intensity gradually decreases and the emission peak shifts from 425 to 510 nm [31]. The surface groups of NCDs introduce trap states with different energy levels resulting in the excitation-dependent emission. The NCDs’ fluorescence quantum yield (FLQY) was calculated to be ~36 % with quinine sulfate (FLQY 54 %) as the reference (see Additional file 1: Figure S1).

**Fig. 3:**
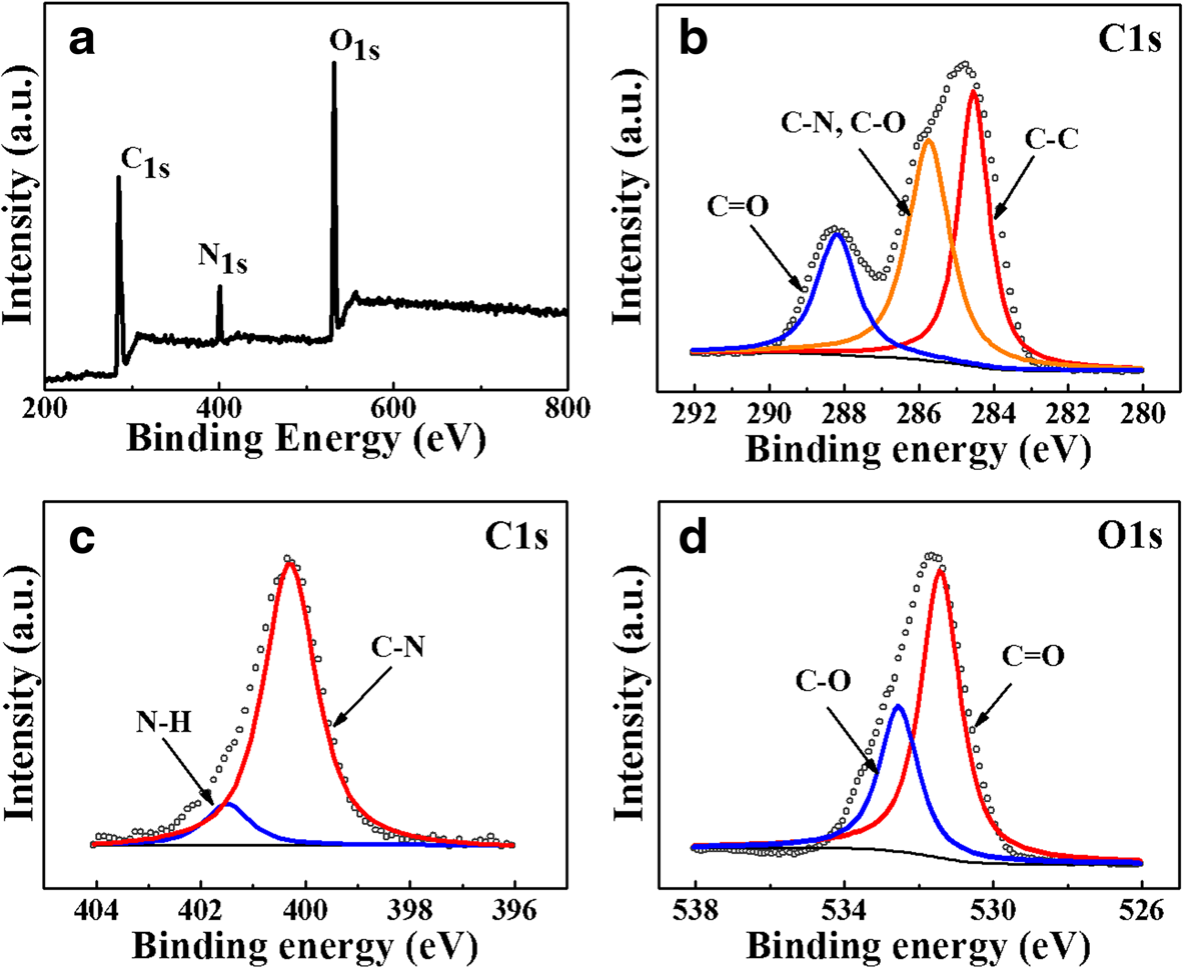
XPS survey scan of NCDs on indium foil. **a** XPS full scan spectrum. XPS high resolution survey scan of (**b**) C1s, (**c**) N1s, and (**d**) O1s region

NCDs were characterized by FT-IR spectroscopy (see Additional file 1: Figure S2). The absorption bands of C–N stretching vibrations at 1182 cm^−1^ confirms the doping of N in the NCDs. Besides, there also observe O–H stretching vibrations at 3000–3400 cm^−1^, C–H stretching vibrations at 2940 cm^−1^, C=O stretching vibrations at 1710 cm^−1^, C–O stretching vibrations at 1408 and 1286 cm^−1^, C–O–C stretching vibrations at 1350 cm^−1^. XPS measurements were performed for the surface elemental analysis of NCDs in Fig. 3a. The full-scan spectrum reveals the existence of carbon (C 1 s, 284.5 eV), nitrogen (N 1 s, 400.5 eV), and oxygen (O 1 s, 531.5 eV). In the expanded XPS spectrum (Fig. 3b), carbon’s 1-s peaks at 284.6, 285.7, and 288.1 eV can be assigned to bonds of C–C, C–N/C–O, and C=O, respectively. Figure 3c illustrates nitrogen 1-s peaks at 400.3 and 401.6 eV suggesting nitrogen exists in the form of C–N and N–H bonds. As shown in Fig. 3d, oxygen 1-s peak exhibits two peaks at 531.7 and 532.8 eV, which are attributed to C=O and C–OH/C–O–C, respectively. It can be concluded that nitrogen-doped CDs have a variety of polar groups including hydroxyl, alkyl, and carboxyl [32].

**Fig. 4:**
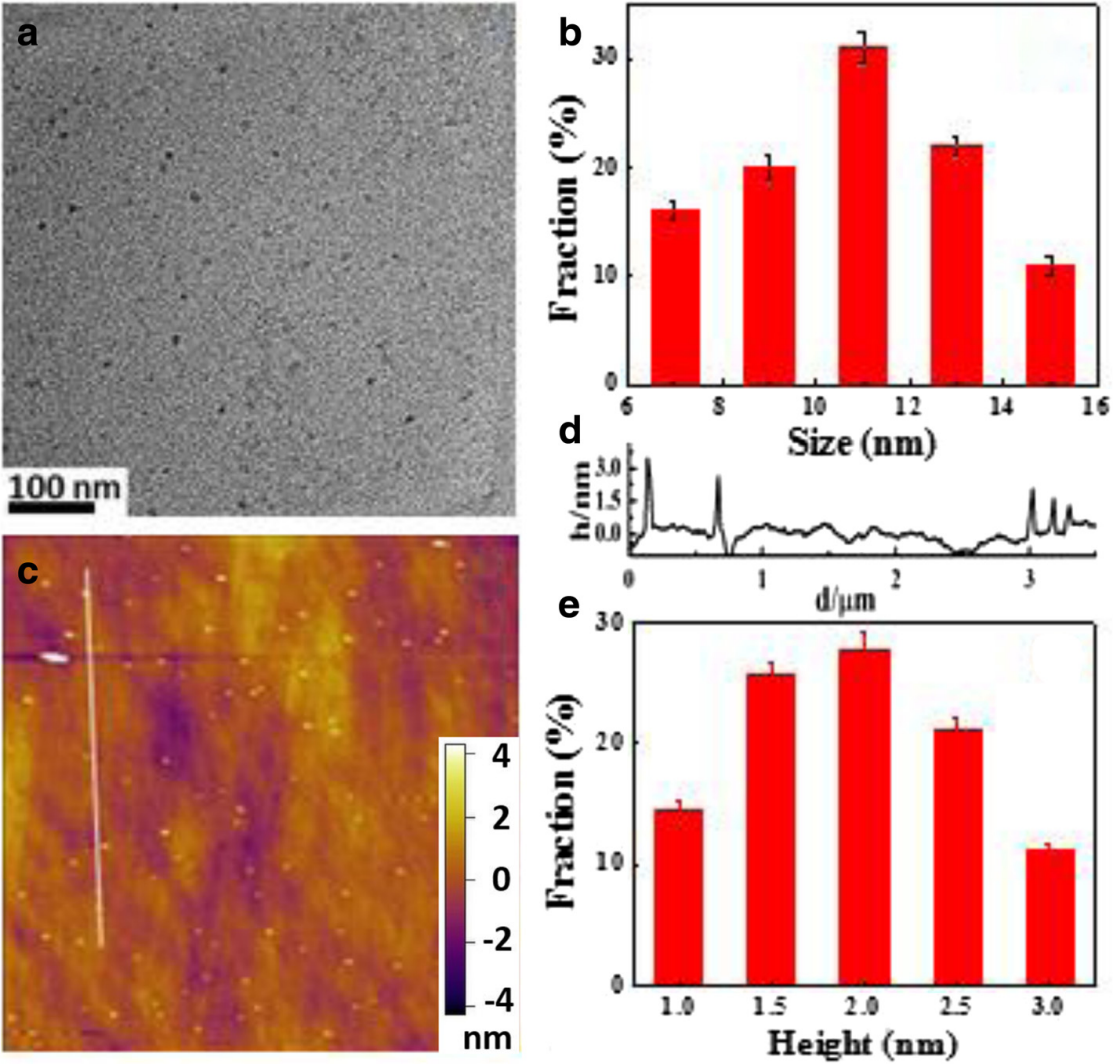
**a** TEM image of NCDs. **b** Particle size distribution of NCDs. **c** AFM image of NCDs deposited on silicon substrate. **d** Height profile along the white line in (**c**). **e** Height distribution of NCDs

The morphology of NCDs was characterized by TEM and AFM in Fig. 4. The TEM images (Fig. 4a, b) show that NCDs’ sizes are mainly distributed in the range of 7–15 nm with an average size of 10.8 nm. The AFM image (Fig. 4c–e) depicts that the topographic height of the NCDs is in the range of 1–3 nm with main distribution at 2 nm. It suggests the NCDs have the cylindrical-like structure (similar to small pieces of graphitic layer stacking) [32]. Raman spectroscopy was used to analyze the relative intensity ratio about 1.07 of the D-band (1342 cm^−1^) and G-band (1531 cm^−1^) (see Additional file 1: Figure S3).

**Fig. 5:**
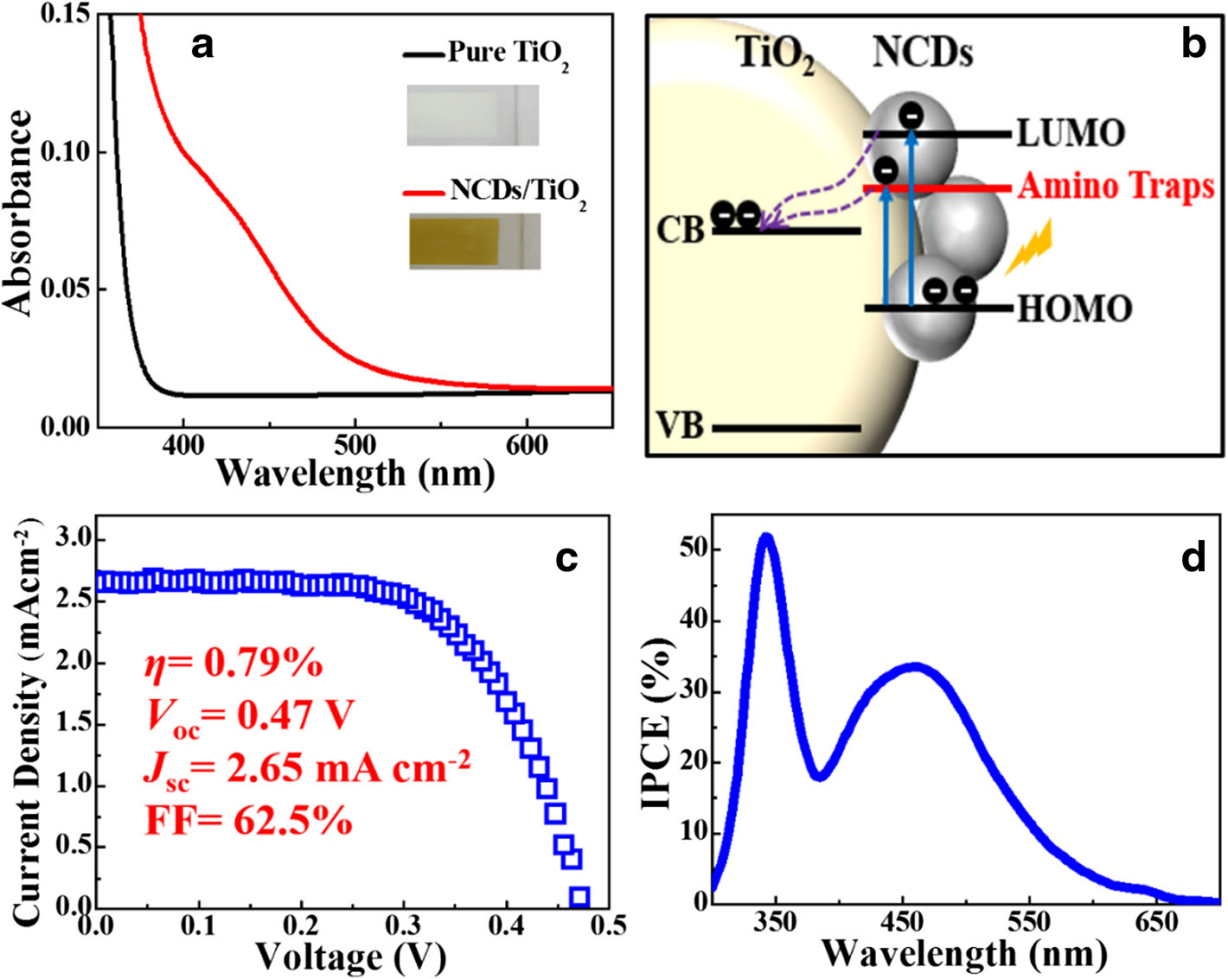
**a** UV–vis DRS spectra of TiO_2_ and NCD/TiO_2_ photoanode. *Insets* are the photographs of TiO_2_ and NCD/TiO_2_ photoanode. **b** A proposed model of photoinduced electron transition between NCDs and TiO_2_. **c** Current density–voltage characteristics and (**d**) IPCE action spectrum of NCD-sensitized solar cells

The enhanced visible-light absorption delivers a message that NCDs could be an attractive interface material integrated with porous TiO_2_ for QDSCs. As a demonstration, NCQDs were combined with anatase TiO_2_. Figure 5a describes UV–vis diffuse reflectance spectrum of TiO_2_ (inset: NCDs/TiO_2_ photoanode). It could be seen that NCD/TiO_2_ photoanode gains much more enhanced absorption in visible region ranging from 400 to550 nm than the pure TiO_2_ photoanode. It should be helpful to apply for photosensitizers. Furthermore, the PL spectrum of NCDs, TiO_2_, and NCDs/TiO_2_ were also studied to confirm the phenomena (see Additional file 1: Figure S4). Under visible light excitation of 420 nm, pure TiO_2_ has negligible PL emission while NCDs exhibit a strong and wide emission peak centered at 550 nm. However, it is interesting to see that the emission of NCDs/TiO_2_ greatly weakened. It indicates that TiO_2_ impedes the recombination of photogenerated electrons and holes in excited NCDs and absorbs the photogenerated electrons. The schematic diagram in Fig. 5b presents the photoinduced electrons transition between NCDs and TiO_2_. Compared to N-free CDs, the N-dopant of NCDs can introduce an additional energy level between π of carbon and π* of oxygen. Once excited, NCDs can absorb the relative low-energy photons at visible light range and generate the photoexcited electrons. As a result, there are more electrons transferring to the conducting band of the TiO_2_, resulting in the higher current density. To investigate the potential application in photovoltaics, we fabricated NCDs as sensitizers for QDSCs. Figure 5c shows the current density–voltage (J-V) curve of NCDs sensitized solar cells under AM 1.5 G one full sun illumination. The solar cell has a *J*_sc_ of 2.65 mA cm^−2^, a *V*_oc_ of 0.47 V, a FF of 62.5 %, and overall PCE of 0.79 %. To our best knowledge, the PCE is the highest value in the reported QDSCs based on CDs. Incident photon-to-electron conversion efficiency (IPCE) is plotted in Fig. 5d. It was found that the overall photocurrent response closely matches the corresponding absorption spectrum of NCD-sensitized TiO_2_ film in Fig. 5a. IPCE value is over 10 % between 400 and 550 nm. It is noting that the highest value of IPCE even approaches 34 % for NCDs.

## Conclusions

In summary, we have developed “green” quantum dot solar cells based on nitrogen-doped carbon dots. The optimal NCD-based solar cells achieve a PCE of 0.79 % with *J*_sc_ of 2.65 mA cm^−2^, *V*_oc_ of 0.47 V, and FF of 62.5 %. The obtained PCE is the highest value in the reported QDSCs based on CDs. The study demonstrates that the further optimization of nitrogen dopant can gain a better conversion efficiency of NCD-based solar cells.

## Additional file

Additional file 1:
**Fluorescence quantum yields and characterizations ofnitrogen-doped carbon dots.** (DOCX 87 kb)
