# Supplementary material for: Nitrogen-Doped Carbon Dots for “green” Quantum Dot Solar Cells
Source: Nanoscale Res Lett. 2016 Jan 19;11:27. doi: 10.1186/s11671-016-1231-1 (PMC4717124; doi:10.1186/s11671-016-1231-1)
Supplement: Additional file 1: — Fluorescence quantum yields and characterizations ofnitrogen-doped carbon dots. (DOCX 87 kb) [file 11671_2016_1231_MOESM1_ESM.docx]

**Supporting Information for**

**Nitrogen-doped carbon dots for ‘green’ quantum dot solar cells**

Hao Wang,^1^ Pengfei Sun,^1^ Shan Cong,^1^ Jiang Wu,^2^ Lijun Gao,*^1^ Yun Wang,^1^ Xiao Dai,^1^ Qinghua Yi^1^ and Guifu Zou*^1^

^1^College of Physics, Optoelectronics and Energy & Collaborative Innovation Center of Suzhou Nano Science and Technology, Soochow University, Suzhou, 215006, P.R.C. China.

^2^Department of Electronic and Electrical Engineering University College London, Torrington Place, London, UK

*corresponding authors: gaolijun@suda.edu.cn; [zouguifu@suda.edu.cn](mailto:zouguifu@suda.edu.cn)

Measurement of fluorescence quantum yields: The FLQY of the as-prepared NGCDs was measured by comparison of their wavelength integrated intensity to that of the standard quinine sulfate. The optical density was kept below 0.1 to avoid inner filter effects. The quantum yields of these oxidized products were calculated using

$$\Phi_{\begin{aligned} S \\ \end{aligned}}=\Phi_{\begin{aligned} R \\ \end{aligned}}\left( \frac{A_{R}}{A_{S}} \right)\left( \frac{I_{S}}{I_{R}} \right)\left( \frac{\eta_{S}^{2}}{\eta_{R}^{2}} \right)$$

where $\Phi$ is the quantum yield, *I* is the integrated intensity, *A* is the optical density and $\eta$ is the refractive index of the solvent. The subscript *S* refers to the sample and *R* refers to the referrence. Quinine sulfate was chosen as the reference, whose quantum yield is 54% in 0.1M sulfuric acid solution.


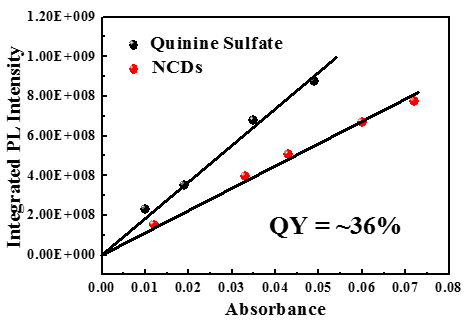


**Fig. S1** Integrated PL intensity of NCDs with different absorbance. Quinine sulfate is used as a reference.


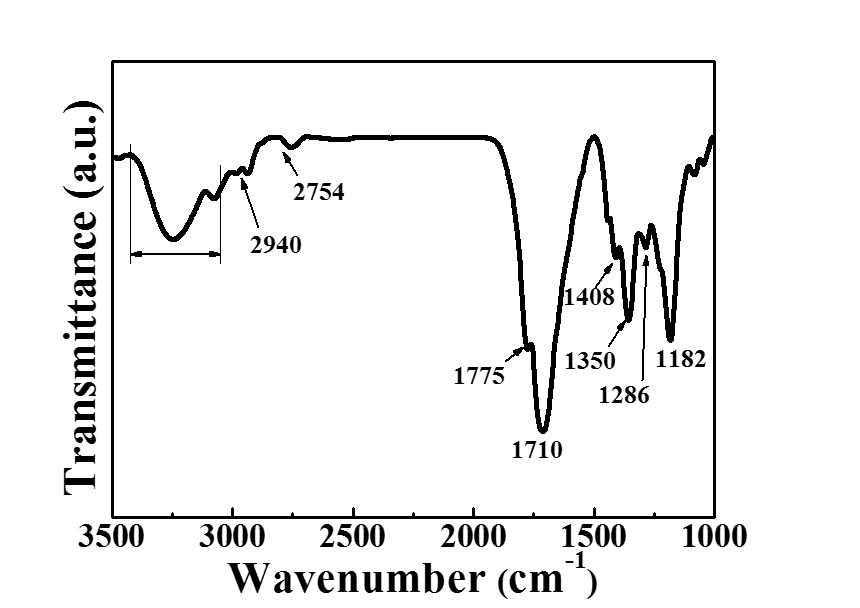


**Fig.S2** FTIR spectrum of the optimal NCDs.


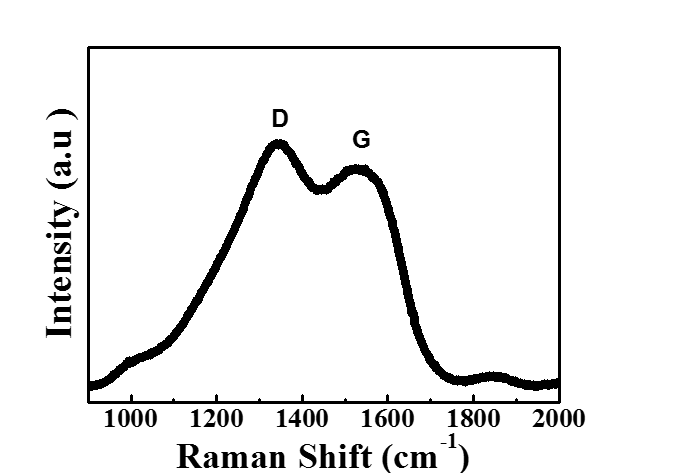


**Fig.S3** Raman spectrum of NCDs


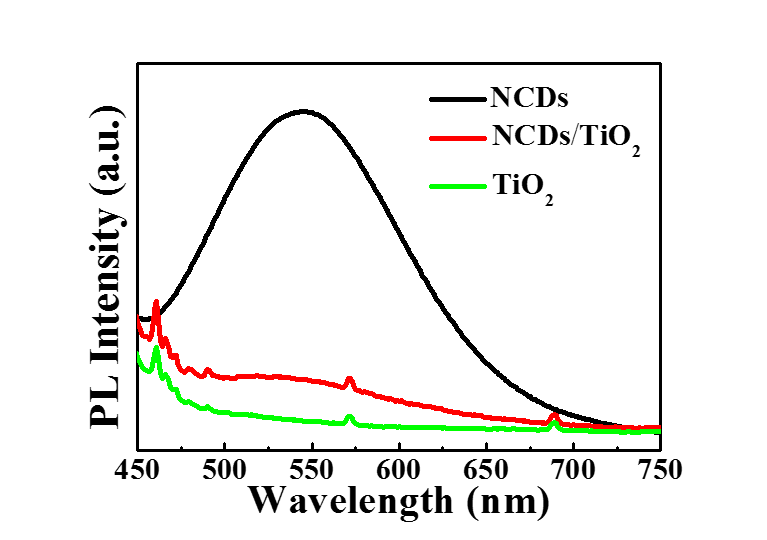


**Fig. S4** PL spectra of NCDs, NCDs/ TiO_2_, and TiO_2_ under excitation of 420 nm wavelength.
